# Affibody molecules as engineered protein drugs

**DOI:** 10.1038/emm.2017.35

**Published:** 2017-03-24

**Authors:** Fredrik Y Frejd, Kyu-Tae Kim

**Affiliations:** 1 Affibody AB, Solna, Sweden; 2 Department of Immunology, Genetics and Pathology, Uppsala University, Uppsala, Sweden; 3 AbClon Inc., Guro-gu, Seoul, Republic of Korea

## Abstract

Affibody molecules can be used as tools for molecular recognition in diagnostic and therapeutic applications. There are several preclinical studies reported on diagnostic and therapeutic use of this molecular class of alternative scaffolds, and early clinical evidence is now beginning to accumulate that suggests the Affibody molecules to be efficacious and safe in man. The small size and ease of engineering make Affibody molecules suitable for use in multispecific constructs where AffiMabs is one such that offers the option to potentiate antibodies for use in complex disease.

## Introduction

Advances in protein engineering technology in the late 90s and early 2000 allowed the generation of several non-antibody protein scaffold formats.^1, 2^ A few of the scaffolds have reached early clinical development stage,^3^ and the Affibody molecule class is among those. In this review, recent advances of the Affibody technology will be reviewed with an emphasis on translational research.

In 1997, Nord *et al.*^4^ demonstrated that a small three-helical subdomain of a natural receptor for the Fc-portion of IgG could be utilized as a general scaffold for diversity. By randomizing amino acids on two of the three helices, large libraries can be constructed, from which potent binders can be isolated by a variety of display methods,^5, 6, 7^ and the scaffold has been engineered for improved synthesis and solubility.^8^ Affibody molecules can be selected to a large variety of different proteins, and can be functionalized with genetic fusions to protein modules^9, 10^ or by chemical conjugation to toxic molecules,^11^ reviewed in Löfblom *et al.*^12^

The Affibody molecule is very robust, and has generated useful biotech tools, for example, as the alkali-resistant active component in MabSelect SuRe for affinity purification of mAbs (MabSelect SuRe, Catalog number 17-5438-01, GE Healthcare, Chicago, IL, USA) or as part of a hot start PCR kit, where the Affibody molecule improves the fidelity of the DNA polymerase by binding to it at ambient temperature, and by surviving a large number of heat cycles (Phusion Hot Start II DNA Polymerase, Catalog number: F549L, Thermofisher Scientific, Waltham, MA, USA). The potential for translation to clinical use is being explored and can be categorized in engineered molecules for diagnostic imaging, use for improving therapeutic outcome by providing guidance during surgery, and therapeutically active molecules to be used as drugs.

## Engineered Affibody molecules for diagnostic imaging

Imaging is an important tool to identify, characterize and monitor tumors. There are several metabolic markers that have been developed (the most used one is fluorodeoxyglucose-positron emission tomography (FDG-PET)^13^), but a scarcity of tumor receptor-specific tracers. Compared with the other protein based affinity tools (for example, monoclonal antibodies and their fragments), Affibody molecules have very small size and hence have favorable properties for diagnostic imaging.^14^ More specifically, a molecular size below 10 kDa allows more rapid extravasation from blood vessels and penetration into tissue, allowing for rapid reach of tumor targets.^15^ Molecules below ~60 kDa have short plasma half-lives as they are cleared via renal filtration.^16^ Altogether, these properties are favorable for clinical imaging use, allowing for high-contrast images within hours after administration of the tracer.

Affibody molecules specific for tumor associated targets have been generated and characterized preclinically. One of the first targets to be explored was HER2,^17^ and the generation of a high affinity (22 pM) Affibody molecule to HER2(^7^) allowed for initial characterization of this molecular class with regards to different valencies and affinities, as well as various nuclide-labeling methods, reviewed in refs 14, 18. Interestingly, it was shown that a monomer Affibody had the same magnitude of tumor uptake as a dimeric higher affinity version, suggesting that small size is of high importance.^19, 20^ It was furthermore shown that high affinity (pM KD) is an important feature for detecting low receptor expression levels but less so for very high expression levels (nM KD is sufficient).^21^

## Clinical receptor quantification *in vivo*

To test the feasibility of targeted detection of tumor HER2 expression in humans, a high affinity HER2-binding Affibody molecule was chemically synthesized and derivatized with 1,4,7,10-tetraazacyclododecane- N,N',N'',N'''-tetraacetic acid (DOTA) for complexation of radiometals. The subsequent clinical study demonstrated for the first time that a non-immunoglobulin derived, non-peptide alternative scaffold protein could target and visualize tumors in humans.^22^ The contrast was very high and allowed tumor detection after injection using single photon computed tomography (SPECT) and an ^111^In-labeled Affibody molecule. The signal was further improved when using positron emission tomography (PET)-imaging and ^68^Ga-labeled Affibody, suggesting broad usefulness of the technology for molecular imaging.^22^ To further investigate the clinical utility of HER2 imaging, a larger study was prepared. To facilitate large scale good manufacturing practice (GMP)-production, the Affibody molecule was now produced recombinantly and denoted ABY-025.^8, 23^ In a first study, ability to detect HER2 expression as early as 2–3 h after injection was demonstrated in a small group of patients using ^111^In-labeled Affibody molecule. Brain metastases could be detected as well as metastases near the kidney, the primary excretion organ.^24^ In a second study, GMP-production of ^68^Ga-labeled ABY-025 was established^25^ and investigated for PET-imaging. This methodology allows for quantification of the uptake and allows the use of the Affibody molecule for determination of the HER2 expression levels. Today, the standard biopsy immunohistochemistry Herceptest^26^ is used for characterization of patients, but the drawback is obviously that the information is limited to the one metastasis that could be biopsied. A global imaging quantification would allow for a more precise determination of receptor status in all lesions and thus provide for better guidance of treatment. Indeed, Sörensen *et al.*^27^ could demonstrate a very good correlation between signal intensity and the control immunohistochemistry score in all metastases tested. Furthermore, conversion from HER2 positive primary tumor to HER2 negative metastases, and vice versa was determined, as well as conversion from HER2 negative tumor to heterogeneous HER2 expression levels in different metastases in the same patient.^27, 28^ In 3 of 16 patients, treatment was changed as a direct consequence of the new knowledge of the HER2-status. If the results from these studies are confirmed in later larger trials, ABY-025 may well change the way patients with HER2 positive tumors are treated in the future.

## Towards intraoperative imaging

Epidermal growth factor receptor (EGFR) is a protein in the same receptor family as HER2, which is overexpressed in many cancers, especially certain brain cancers.^29^ Like HER2, the receptor is a growth factor receptor, and as overexpression thus promotes tumor growth,^30^ it is in principle difficult for the tumors to escape EGFR targeted therapies. Affibody molecules for EGFR have been generated and as there are quite high levels of EGFR-expression also in normal tissues like skin and liver, binders were isolated that would cross react with murine EGFR to allow for more accurate preclinical studies to be performed.^31^ This has allowed preclinical tumor-targeting studies in mice in presence of endogenous background levels of EGFR, and feasibility of high-contrast tumor images in tumor bearing mice has been demonstrated.^32, 33, 34^ Detailed studies on the composition of a peptidic chelator fused to the EGFR-specific Affibody molecule revealed a marked difference in excretion pattern after a change of just a few amino acids.^35^ Inspired by the early tumor-targeting results, Gong *et al.*^36^ labeled the EGFR-specific Affibody molecule with a near infrared fluorescent probe, to visualize tumors without radioactivity. The EGFR-specific Affibody molecule bound to and was taken up by EGFR-expressing A431 cancer cells *in vitro*, and in contrast to the natural ligand, EGF, the Affibody molecule did not activate EGFR signaling pathways. When intravenously injected in tumor bearing nude mice, the tumors could be detected as early as one hour after injection, and images of dissected tissue sections confirmed high uptake in the tumor, and liver, both displaying high EGFR-expressing levels, and kidney, the main excreting organ. In combination with a HER2-targeting fluorescently labeled Affibody molecule, it was further shown that the EGFR-specific Affibody molecule could discriminate between EGFR- and HER2-expressing tumors and that the EGFR-specific molecule is a promising candidate for molecular imaging of EGFR-expressing tumors.^36^

The concept has since then been further developed by Pogue and colleagues,^37^ who recognized that a challenge during surgical resection of malignant gliomas is to remove all tumor tissue while sparing as much as possible of the healthy brain tissue surrounding the tumor lesion, to preserve brain function. Today, to better visualize the tumor border, a prodrug is orally administered that is metabolized to a fluorescent dye in the metabolically active cancer cells and surgery is performed using detection of the emitted UV-light. A drawback of UV light is however very short tissue penetration, and that this wavelength is quenched by blood. In addition, it works well only with the most metabolically active lesions. Pogue and colleagues^37^ investigated if one could use the fact that many gliomas have very high expression levels of EGFR. With the hypothesis that a small sized targeting agent would provide better tissue penetration and tumor border contrast than a larger molecule, they compared the EGFR-specific clinically used monoclonal antibody cetuximab^38^ with an EGFR-specific Affibody. Both molecules showed accumulation in orthotopically growing brain tumors in mice. There was however a better contrast in the proliferative tumor border with the smaller Affibody compared with the antibody that seemed to be more restricted to the central part of the tumor where the blood brain barrier was more disrupted (7 kDa vs 150 kDa), and the authors concluded that the Affibody molecule showed the highest potential for use as a clinical imaging tool.^37^ To pave way for a cost efficient potential clinical program, it was demonstrated that even a corresponding microdose of an EGFR-specific Affibody molecule, denoted ABY-029, was sufficient for delineation of human glioma xenografts in nude rats.^39^ It was furthermore shown that ABY-029 imaging outperformed the standard 5-aminioleuvulinic acid agent in a direct comparison in orthotopically implanted rats but also that the overall imaging analysis could be further improved if ABY-029 was added to the standard agents and both signals used.^40^ To test the utility of ABY-029 and to address the challenge of translation of novel agents to the clinic with very limited funding, ABY-029 has been synthesized chemically to support a phase 0 clinical trial to obtain initial characterization of the molecule, supported by a single NIH grant.^41^ The first in man study was recently approved by the FDA and ABY-029 will soon be tested in patients with recurrent glioma (NCT02901925).

## Early stage imaging agents

A number of cancer associated targets have been identified for generation of Affibody molecules, and include targets expressed both directly on the cancer cells and targets that are more associated with the physiology of the tumor. HER3 is the third member of the EGFR family described above, and is an important driver of oncogenesis as a heterodimer with other family receptors.^42^ In contrast to EGFR and HER2, HER3 is not massively overexpressed in tumors, and there are substantial levels of normal tissue expression. An Affibody molecule that is cross reactive with the murine target was therefore isolated to study targeting and uptake in relevant models.^6, 43^ As the expression levels are relatively low, high affinity variants were needed, and affinity maturation resulted in low picomolar binders^44^ that demonstrated good tumor-targeting properties in xenografted mice.^45^ To test the ability to discriminate between high and low HER3 expression, a PET-labeling methodology was established, and it was shown using micro PET that uptake of the tracer was much higher in xenografts with high HER3 expression (BT474, BxPC-3) as compared with the xenografts with low HER3 expression (A431).^46^ To address the heterodimeric nature of HER3 signaling, a novel bispecific format was constructed targeting both HER2 and HER3, and with a half-life extension module in the linker region. The molecule could simultaneously bind both HER2 and HER3 and efficiently blocked ligand-induced phosphorylation, indicating a potential use for therapy.^47^ The molecule is currently being evaluated further for therapeutic applications.

Another target with low tumor expression levels is the IGF1R, making it a challenging candidate for imaging. Tracer requirements are high enough affinity and good enough contrast due to rapid blood clearance to be able to monitor IGF1R, which is reported to be highly important in resistance mechanisms of tumors. Gräslund and colleagues^48^ tested if the Affibody technology could be used and IGF1R binders were isolated. Feasibility of tumor targeting in DU-145 prostate cancer xenografts in mice was shown,^21^ and subsequent studies showed useful contrast between tumor and most other organs.^49, 50^

An interesting class of tumor imaging targets relates to markers that are associated with the physiology of the tumor environment, rather than being directly expressed on the tumor cells. Such markers include CAIX that is upregulated in the hypoxic conditions in solid tumors,^51^ VEGFR2 being a driver for tumoral angiogenesis,^52^ and PDGFR, that is expressed on activated pericytes and fibroblasts in tumor stroma.^53^ Imaging tracers for such markers could provide a more general imaging modality that in principle would be less restricted to tumor cell phenotype. Affibody molecules have been isolated and shown to be able to target tumors having expression of CAIX^33, 54^ and PDGFR.^55, 56, 57^ As expression levels of VEGFR2 are low, and available only in a small cell population within the tumor, efforts have been directed towards enhancing the affinity of the VEGFR2-specific Affibody molecule. Here, two Affibody molecules binding to two distinctly different target epitopes were combined, and a biparatopic format was engineered, with substantially improved binding affinity.^58^ In addition, the resulting biparatopic molecule blocked ligand receptor interaction and inhibited angiogenic sprout formation of endothelial cells as efficiently as the antibody ramucirumab, which is approved for treatment of metastasized lung, colorectal and gastric cancer.^59^ This suggests that Affibody molecules could be used for therapeutic intervention, and will be discussed below.

## Towards therapeutic application of Affibody molecules

Affibody molecules have the ability to bind protein targets with high affinity and selectivity. In contrast to antibodies that have Fc, however, they lack half-life extension and effector function modules. Therapeutic action can thus either be directly carried out by blocking ligand receptor interactions, as shown by antibodies in inflammatory conditions,^60, 61^ or by functionalizing the Affibody molecules to have long half-lives and toxic payloads. One of the first therapy examples using this approach was the use of a HER2-targeted Affibody molecule,^7^ which had shown excellent tumor uptake in mice,^62^ and is in development for metastasized breast cancer.^24, 27^ The diagnostic agent however, had relatively high kidney uptake levels and could not be directly used for targeting of potent toxic payloads. Kidney uptake of small proteins is a normal physiologic mechanism, and the size cut off is around 60 kDa, just below the size of serum albumin, which is a major plasma protein with very long half-life.^63^ To solve the problem of kidney uptake, a small engineered albumin-binding domain (ABD) was genetically fused to the HER2-specific Affibody molecule. The resulting fusion protein indeed displayed very long circulatory half-life with increased dose to the tumor, substantially reduced kidney uptake and demonstrated potent antitumor effect in a micrometastatic model of breast cancer when conjugated with the therapeutic radionuclide ^177^Lu.^9^ The construct was further improved using an affinity matured ABD with femtomolar-binding affinity,^64^ an optimized HER2-binding Affibody molecule,^8^ and site-specific labeling of the radionuclide by using a cysteine as chemical handle, further lowering the kidney uptake and increasing tumor to normal organ ratios.^9^ The albumin-binding domain has subsequently been optimized and deimmunized,^65^ and has been demonstrated to enhance the half-life in several different species^66^ and recently also in humans (NCT02690142).

Radionuclide payloads have several advantages, but also some drawbacks in terms of logistics and some potential side effects. Other payloads have been investigated with Affibody molecules, and an attractive approach is to use a protein toxin, since this allows for a manufacturing process based on a single polypeptide chain. Pseudomonas exotoxin is a well characterized toxin that in a truncated version (PE38) has been fused to antibody fragments to create a targeted immunotoxin. This was tested also for the HER2-targeting Affibody molecule,^67^ and the resulting fusion protein was shown to completely eradicate large BT474 tumors in xenograft models.^10^ The immunotoxin had a short half-life to minimize potential side effects, but speculating that longer half-life would enhance the effect of the construct, Graslund and co-workers^68^ constructed a triple functional targeted toxin by fusing a deimmunized PE38 to the albumin-binding domain described above and to the targeting HER2 Affibody molecule. The fusion protein reacted with serum albumin, and IC50s from 6 to 300 pM were reported in cell assays, where a short incubation of 10 min resulted in substantial cell killing. Also Guo *et al.*^69^ demonstrated the ability to fuse PE38 to ABD and HER2 to enhance potency, and they found that in comparison to immunotoxin without ABD, the half-life was improved almost 25 fold, and with improved antitumor effects in NCI-N87 tumor xenografts.

A general problem when using small toxic payload molecules conjugated to carrier proteins is the toxic side effects that occurs where the drug is degraded or, with radioactive payloads, in the bone marrow due to high blood exposure. Pre-targeting is a way to solve this problem by separating the targeting moiety and the effector domain in time. This is especially attractive with the Affibody molecules as they have both good tumor targeting and rapid kinetics.^14, 27^ In principle, the two step targeting approach could be carried out within a day, which substantially would improve future clinical routine. In a first test, Tolmachev and colleagues^70^ tested an anti-HER2 Affibody-based peptide nucleic acid with a complementary hybridization probe that showed low kidney uptake and specific high affinity binding on HER2-expressing cells *in vitro* with substantial tumor uptake in HER2-expressing xenografts *in vivo*. The uptake was specific and tumor to blood levels of 50:1 and tumor to kidney of 2:1 suggests potential for radioimmunotherapy.^54^ In a second study, trans-cyclooctene (TCO) and tetrazine were tested as a system to enhance the accumulation of radiometals in tumor xenografts over kidneys. TCO was conjugated to a HER2-targeting Affibody molecule that was shown to bind to HER2-expressing cells. A DOTA-derivatized tetrazine was then labeled with radiometals and shown to localize in tumors that were pretargeted with the Affibody-TCO construct. Compared with a directly labeled tracer, pre-targeting resulted in a 56-fold reduction of renal uptake with two times more activity in the tumor as compared with the kidney and 50-fold more activity in the tumor as compared to blood.^71^ In summary, both the peptide nucleic acid and the biorthogonal chemistry approaches allowed administration of the effector module already 4 h after injection of the targeting agent, and pre-targeting using Affibody molecules with rapid kinetics could be an attractive approach towards more efficient payload therapy.

Blocking of a disease causing agent by binding to it is a tractable therapeutic modality, as it does not require modification of the binding molecule. Monoclonal antibodies have demonstrated the feasibility in several inflammatory conditions, but in some diseases with very high target concentrations there is a limitation in the amount of antibodies that can be delivered in the one milliliter volume that can be administered subcutaneously without discomfort. Typically, very high antibody concentrations are needed (>100 mg ml^−1^), and if that is not sufficient, intravenous infusions are required, which means hospital visits and limitations for patients living in remote areas. Here, the Affibody technology, which given the small molecular size offers the potential to increase the dose 10-fold in the same volume, would be attractive to employ. In several rare autoimmune diseases, the complement system is aberrantly activated. One central part of the complement system is C5, and eculizumab is a C5 blocking monoclonal antibody approved for the treatment of paroxysmal nocturnal hemoglobinuria (PNH) and atypical hemolytic uremic syndrome (aHUS), two rare disease caused by an autoimmune activation.^72, 73^ The drug is dosed by infusion at very high doses, up to 1200 mg every second week. An alternative with potential for subcutaneous home use administration and with less costly production would be attractive. Affibody molecules specific for C5 were generated using phage display technology, and candidates that demonstrated cross reactivity with both rodent and cyno and that had affinity in the low to subnanomolar range were selected. The molecules were engineered in different formats for tailored kinetic properties, including PEGylation, Fc-fusion or fusion to ABD for albumin binding. An ABD-fused anti-C5 Affibody molecule that could inhibit C5-dependent hemolysis *in vitro* and potently block C5 *in vivo* in a Zymosan-induced peritonitis mouse model was recently tested in healthy volunteers (NCT02083666).^74^

Another target where very high antibody doses are used in clinical trials is Abeta in Alzheimer's disease (AD). Several antibodies employed in current AD clinical trials are administered at very high doses corresponding to 300–800 mg per patient (80 kg), which requires intravenous infusion every three weeks or so.^75, 76^ Given the notion of a life-long preventive treatment and the logistics of the high number of potential AD patients that would thus require dosing, it would be highly desirable to identify a drug that could be administered at home in an outpatient subcutaneous setting instead of requiring i.v. infusion centers. Affibody molecules specific for Abeta were developed to address this challenge. Abeta is a key component in the development of AD, and is present in several different isoforms.^77^ It is believed that inhibition of the ability of Abeta to aggregate to form plaques could be a major therapeutic opportunity. Stahl and colleagues^5^ isolated an initial set of Abeta binding Affibody molecules, that preferentially bound non-aggregated Abeta. The protein structure was solved using NMR and it was shown that the Affibody molecule ZAbeta3 stabilized a β-hairpin of the monomeric amyloid-β peptide to act as an inhibitor of Aβ fibrillation.^78^ To test if the ZAbeta3 Affibody molecule could act therapeutically *in vivo*, it was tested in a fruit fly model of AD. ZAbeta3 in monomeric and dimeric formats were co-expressed in the brain of fruit flies expressing either Abeta42 or the aggressive variant E22G of Abeta and the life span of the fruit flies was studied. The results were impressive, with completely abolished neurotoxic effects in the flies that received the dimeric Affibody molecule.^79^ To further improve this molecule, it was hypothesized that enhanced affinity was needed. A Staphylococcal bacterial display system was established to thoroughly characterize the Affibody molecules. In addition to functional expression, determination of affinity and pH-sensitivity, effects from truncation of the N-terminal part of the Affibody molecule was investigated.^80^ On the basis of this work and structure information available,^78^ an affinity maturation effort was undertaken using Staphylococcal display to guide selection of improved binders. Two different affinity maturation libraries were designed and subjected to selections. Improved binders could be isolated and the best had a 50-fold improved affinity with around 300 pM KD.^81^ This binder was fused to an ABD and tested *in vivo* in a double transgenic mouse model of AD and shown to efficiently protect the mice from Abeta-induced pathology.^81^

The clinically most advanced Affibody molecule is currently an engineered IL-17-specific ligand trap. IL-17 is a key driving molecule in psoriasis, and moderate to severe psoriasis strongly impacts the quality of patient lives.^82^ To create an IL-17 blocking molecule with a potency superior to the monoclonal antibodies ixekizumab^83^ and secukinumab,^84^ IL-17-specific Affibody molecules were formatted into a small 18 kDa dimeric construct with built in long plasma half-life using a previously reported format.^47^ By simultaneously binding and blocking both subunits of the dimeric IL-17 molecule, the affinity was increased ten-thousand fold to sub-picomolar KD affinity. No adverse findings were reported in preclinical toxicity studies, and the molecule was named ABY-035 and has entered clinical development. Recently, the healthy volunteer dose escalation part of the phase I study was completed and initial results in more than 50 subjects suggest the compound to be safe and well tolerated (NCT02690142). ABY-035 is being evaluated in patients with plaque psoriasis.

## AffiMabs

A recent trend in biologics development is towards creation of multispecific therapeutic constructs, with antibodies leading the way.^85^ A novel class is using antibodies as a basic scaffold, which is then fused to peptides or alternative scaffolds to functionalize the antibody with enhanced properties. This has been shown with small peptides^86^ and recently also with alternative scaffold proteins.^87, 88^ Affibody molecules have been demonstrated to be useful as molecular specifiers for antibodies in several labs, including our own, and more than six different Affibody molecules have been combinatorially fused with antibodies to form functional multispecific proteins called ‘AffiMabs'.^87, 89^ There are various formats of multispecific antibodies described which have quite different structures from the canonical IgG format. In contrast, AffiMabs retains symmetric bi-valency and Fc of common IgGs, and AffiMabs are supposed to have corresponding substantial half-life and stability *in vivo* and facile manufacturability. La Fleur and colleagues showed that it is possible to create pentaspecific antibody constructs using Affibody molecules. They also showed superior *in vivo* therapeutic activity in a xenograft tumor model when administering a bispecific molecule based on the EGFR-specific antibody cetuximab and a HER3-specific Affibody molecule. The bispecific molecule more efficiently inhibited cell growth of the pancreatic cell line BxPC-3 *in vitro* and *in vivo* as compared with the parental cetuximab antibody, which is widely used in cancer therapy.^42, 87^

Nygren and co-workers later investigated if AffiMabs could be employed to target inflammatory disease and soluble disease mediators. They noted that many inflammatory conditions are driven by multiple pathways, and reasoned that it would make sense to block at least two of them simultaneously.^90, 91, 92^ TNF and IL-6 blocking agents are both approved in RA and with similar efficacy (ADACT trial, NCT01119859). It is believed that IL-6 is more important in the systemic part of the disease whereas TNF controls local inflammation.^93^ To address this hypothesis, an IL-6-specific Affibody molecule that is active for blocking IL-6 trans-signaling was isolated. This is important since there is a lot of IL-6 bound to shredded IL-6R in the circulation, which can stimulate any cell (*trans*) that express the co-receptor gp130,^94^ as compared with the action of IL-6 itself, which can only active cells expressing the receptor on their cell membrane (*cis*). The Affibody molecule was fused to N- or C-terminal positions of the heavy or light chain, respectively, of a TNF-specific antibody scaffold derived from adalimumab and characterized for binding and activity. The Affibody molecule was active as fused to all different positions of the antibody, and both parental molecules retained their activity in binding assays. Furthermore, in a growth stimulation TF-1 cell assay, it was shown that the AffiMab was superior in blocking cell activation in comparison with the parental antibody. This was later confirmed *in vivo*, where TNF and IL-6 was used to activate the production of serum amyloid alpha (SAA) in mice. There was pronounced production of SAA in control mice and only partial inhibition using the TNF-specific antibody, in contrast to the AffiMab, where there was robust and complete inhibition of the cytokine induced SAA response.^89^ This suggests that AffiMabs could become useful in diseases where multiple pathways are activated, and further investigation is warranted. The IL-6/TNF-specific AffiMab is currently in preclinical development (http://www.abclon.com).

## Conclusion

**Figure 1:**
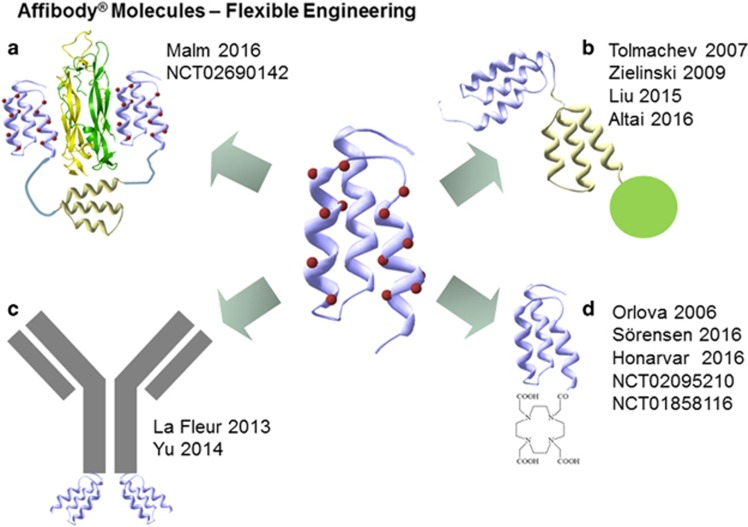
Affibody molecules can be engineered in different formats. (**a**) Hetero- or homospecific bivalent and half-life extended, (**b**) monospecific half-life extended with payload, (**c**) as additional molecular specifier in multispecific antibody based formats and (**d**) as monospecific carrier of a payload with rapid kinetics and high contrast.

In conclusion, Affibody molecules can be used as tools for molecular recognition in diagnostic and therapeutic applications. There are several preclinical studies reported on diagnostic and therapeutic use of this molecular class of alternative scaffolds, and early clinical evidence is now beginning to accumulate that suggests the Affibody molecules to be efficacious and safe in man. The small size and ease of engineering make Affibody molecules suitable for use in multispecific constructs (Figure 1) where AffiMabs is one such that offers the option to potentiate antibodies for use in complex disease.
